# Diclofenac Removal by Actinobacterial Strains and Ecotoxicological Responses

**DOI:** 10.3390/ijms27219952

**Published:** 2026-11-06

**Authors:** Victoria Guadalupe Gonzalez Holc, María Alejandra Small, Gonzalo Arnau, Sylvie Lautru, Jean-Luc Pernodet, Juan Daniel Aparicio, Marta Alejandra Polti

**Affiliations:** 1 Planta Piloto de Procesos Industriales Microbiológicos (PROIMI), CONICET, Av. Belgrano y Pasaje Caseros, Tucumán 4000, Argentina; vgonzalezholc@conicet.gov.ar (V.G.G.H.); alejandrasmall@conicet.gov.ar (M.A.S.); garnau@conicet.gov.ar (G.A.); jdaparicio@conicet.gov.ar (J.D.A.); 2 Université Paris-Saclay, CEA, CNRS, Institute for Integrative Biology of the Cell (I2BC), 91198 Gif-sur-Yvette, France; sylvie.lautru@i2bc.paris-saclay.fr (S.L.); jean-luc.pernodet@i2bc.paris-saclay.fr (J.-L.P.); 3 Facultad de Bioquímica, Química y Farmacia, Universidad Nacional de Tucumán, Ayacucho 491, Tucumán 4000, Argentina; 4 Facultad de Ciencias Naturales e Instituto Miguel Lillo, Universidad Nacional de Tucumán, Miguel Lillo 205, Tucumán 4000, Argentina

**Keywords:** *Streptomyces*, emerging contaminant, ecotoxicity, pharmaceutical, diclofenac

## Abstract

Diclofenac (DCF) is a widely detected pharmaceutical whose persistence and biologically active transformation products raise environmental concern. This study evaluated DCF tolerance and removal by actinobacterial strains and integrated chemical performance with plant- and earthworm-based ecotoxicological responses. Phenotypic screening at 1, 5, and 10 g L^−1^ DCF revealed strain- and concentration-dependent responses in growth, sporulation, and pigment production, with *Streptomyces* sp. E5N50 and *Streptomyces* sp. Z2 selected for further study. In liquid assays, after 5 days of incubation, only low biomass production was observed when 50 mg L^−1^ DCF was supplied without glucose, relative to glucose-containing controls. E5N50 showed modest DCF removal (approximately 23–26%), while Z2 achieved 90.8% in glucose-supplemented medium and about 70% when glucose and ammonium were supplied. A seven-day time-course study with *Streptomyces* sp. Z2 showed that DCF removal continued after biomass had reached its maximum, suggesting that the process was not exclusively growth-associated. Ecotoxicological responses were organism- and endpoint-dependent. The glucose + DCF + Z2 treatment, associated with the highest DCF removal, significantly increased tomato radicle elongation, whereas glucose + ammonium + DCF + Z2 produced the most favorable *Eisenia fetida* response, with significantly reduced body-mass loss and increased coelomocyte abundance despite the lower DCF removal removalobserved in this treatment. Overall, Z2 exhibited potential for DCF removal under defined laboratory liquid-culture conditions, and the combined chemical and biological assessment highlighted that parent-compound removal and ecotoxicological improvement provide complementary measures of remediation performance.

## 1. Introduction

Diclofenac (DCF) is a non-steroidal anti-inflammatory drug widely used in human and veterinary medicine (Figure S1). After use, DCF and its metabolites enter domestic, hospital, industrial, and agricultural waste streams through excretion, washing of topical formulations, and improper disposal. Conventional wastewater-treatment plants were not specifically designed to eliminate pharmaceuticals; consequently, DCF is repeatedly introduced into surface waters, groundwater, soils, and sediments through treated effluents, sewage sludge, and irrigation with reclaimed water [1,2,3].

Although DCF can undergo photochemical and biological transformation, continuous environmental inputs make it functionally pseudopersistent. Its biological activity and the formation of transformation products (TPs) are particularly relevant because some TPs retain structural features of DCF, persist in aquatic systems, or exhibit toxicity comparable to or greater than that of the parent compound [4,5]. Some of de reported TPsinclude 4′-hydroxydiclofenac, 5-hydroxydiclofenac, 3′-hydroxydiclofenac, 4′,5-dihydroxydiclofenac, and quinone imine derivatives [6]. Adverse effects have been reported in aquatic organisms, birds, plants, and soil biota, and DCF can be accumulated or transformed by exposed organisms [7,8,9,10]. Therefore, remediation studies should evaluate both chemical removal and biological effects.

Microbial treatment is a potentially low-impact strategy for removing pharmaceutical residues. DCF transformation has been reported in activated sludge, soil-derived consortia, and individual bacterial isolates, but performance varies markedly with strain, DCF concentration, and the availability of auxiliary carbon sources [11,12,13,14,15]. This variability reflects the distinction among tolerance, adsorption, transformation, mineralization, and growth on DCF. In particular, disappearance of the parent compound cannot be equated with biodegradation unless abiotic loss, sorption, and TPs are appropriately assessed.

Actinobacteria are promising biocatalysts because of their metabolic versatility and capacity to transform structurally complex xenobiotics [16,17]. Nevertheless, DCF removal by this group remains poorly characterized. *Rhodococcus ruber* IEGM 346 transformed DCF at environmentally relevant concentrations [18], whereas *Brevibacterium* sp. D4 removed DCF under acetate-supplemented conditions [11]. Whether other actinobacterial genera exhibit comparable DCF removal capacities, and how carbon and nitrogen availability affect their performance, remain insufficiently explored.

We hypothesized that actinobacterial strains previously selected for their ability to tolerate or transform xenobiotics would differ in DCF tolerance and removal, and that carbon and nitrogen availability would modify these responses. Accordingly, this study (i) screened a collection of actinobacteria for phenotypic tolerance to DCF; (ii) compared biomass production, nutrient consumption, and DCF removal by the most tolerant strains under different carbon and nitrogen regimes; (iii) examined the temporal relationship between growth and DCF disappearance in the best-performing strain; and (iv) evaluated selected plant and earthworm responses to the resulting culture media.

## 2. Results and Discussion

### 2.1. Diclofenac Tolerance by Actinobacterial Strains

Phenotypic tolerance to DCF varied markedly among the actinobacterial strains and decreased as DCF concentration increased (Figure 1 and Figure S2). In DCF-free medium, composite scores ranged from 7 to 9. At 1 g L^−1^ DCF, several strains retained high scores, particularly *Streptomyces* sp. SG1 (9), *Streptomyces* sp. E5N50, *Streptomyces* sp. SG21, and *Streptomyces* sp. Z2 (8), whereas *Streptomyces* sp. S1 showed complete inhibition (score 0). Increasing the DCF concentration to 5 g L^−1^ further differentiated the strains: E5N50 and Z2 retained scores of 8, SG1 and SG21 scored 6, while *Amycolatopsis tucumanensis* AB0, *Streptomyces* sp. ER1, *Streptomyces* sp. MC1, and *Streptomyces* sp. S1 showed no detectable phenotypic response. At the highest concentration tested (10 g L^−1^), Z2 showed the highest composite score (6), followed by SG1 (5), whereas E5N50 decreased to 2. Most of the remaining strains showed little or no growth, sporulation, or pigment production at this concentration.

Overall, Z2, SG1, and E5N50 displayed the greatest tolerance across the DCF concentration range, with Z2 showing the highest composite score at 10 g L^−1^. Differences in composite scores between strains exhibiting apparently similar growth may reflect variations in sporulation and spore pigmentation, which were evaluated independently and were not always clearly distinguishable in the photographic records (Figure S2). Considering their high tolerance at 5 g L^−1^, Z2 and E5N50 were selected for further study.

The simultaneous reduction in vegetative growth, sporulation, and pigmentation indicates that DCF affected both biomass development and differentiation. DCF exposure can modify oxidative balance, membrane permeability, and cell-surface properties in bacteria, although the magnitude and direction of these effects depend on the organism and exposure conditions [15]. In *Streptomyces*, morphological differentiation and specialized metabolism are closely coordinated and respond to environmental stress [19,20]. The observed changes are therefore consistent with broad physiological stress, but they do not identify a specific mechanism. In particular, persistence of pigmentation cannot by itself be interpreted as evidence of antioxidant protection or DCF transformation.

### 2.2. Diclofenac Removal by the Selected Strains

The DCF concentrations used in this study were selected to assess microbial tolerance under stringent conditions (1 to 10 g L^−1^) and compare removal capacities in controlled liquid assays (50 mg L^−1^). As these concentrations exceed those typically detected in environmental waters, further studies at environmentally relevant concentrations and in realistic matrices would help establish the applicability of these findings.

Biomass production, nutrient consumption, and DCF removal differed between *Streptomyces* spp. E5N50 and Z2 and were strongly influenced by medium composition (Figure 2). In the DCF-free glucose + ammonium control, biomass production reached approximately 0.30 g L^−1^ for both strains. Considerably lower biomass values were recorded in the DCF-containing treatments. In glucose + DCF medium, biomass reached approximately 0.07 g L^−1^ for E5N50 and 0.09 g L^−1^ for Z2, whereas only low biomass production was observed when DCF was supplied without glucose. Glucose consumption was high in both strains, reaching approximately 71–76% in E5N50 and 85–89% in Z2 in the DCF-containing glucose treatments. In the DCF-free glucose + ammonium controls, glucose consumption was approximately 74% for E5N50 and 97% for Z2.

Ammonium consumption also differed between strains and treatments. In the DCF-free glucose + ammonium medium, E5N50 and Z2 consumed approximately 18 and 17% of the supplied ammonium, respectively. In the presence of DCF, E5N50 showed only limited ammonium consumption in the ammonium + DCF condition, whereas Z2 consumed approximately 17% when glucose, ammonium, and DCF were simultaneously present.

The largest difference between the strains was observed for DCF removal. E5N50 showed relatively similar removal efficiencies among the DCF-containing treatments, ranging from approximately 23 to 26%. In contrast, Z2 showed a pronounced dependence on nutritional conditions. DCF removal was approximately 32% when DCF was supplied without glucose and approximately 26% in ammonium + DCF medium, but increased to 90.8% in glucose + DCF medium. When both glucose and ammonium were supplied, Z2 removed approximately 71% of the initial DCF. Thus, glucose supplementation produced the greatest increase in DCF removal by Z2, whereas the addition of ammonium together with glucose was associated with a lower removal efficiency than glucose alone.

Although Z2 exhibited the highest phenotypic tolerance and DCF removal (90.8%), E5N50 also showed considerable tolerance but achieved only 23–26% removal, suggesting that tolerance does not necessarily reflect biotransformation capacity. Therefore, strain selection for bioremediation applications should consider both phenotypic tolerance and actual DCF removal efficiency.

The absence of net growth with DCF as the sole carbon source does not support its use as a primary growth substrate by either strain under the conditions evaluated. By contrast, the enhanced disappearance of DCF when glucose was metabolized is consistent with a cometabolic contribution. However, further studies are needed to confirm this hypothesis. An auxiliary carbon source can supply energy and reducing equivalents for enzymes that fortuitously transform a non-growth substrate. Similar nutrient-dependent responses have been observed in bacterial DCF-removal systems, although the effect of a given co-substrate is strain-specific [13,14,15].

### 2.3. Growth and DCF Removal Kinetics of Streptomyces *sp.* Z2

The time-course experiment revealed distinct patterns of biomass production and DCF disappearance in *Streptomyces* sp. Z2 depending on nutrient availability (Figure 3). In the DCF-free glucose control, biomass increased rapidly from approximately 0.16 g L^−1^ at the beginning of the experiment to approximately 0.62 g L^−1^ after 24 h and reached a maximum of approximately 0.70 g L^−1^ on day 2. Biomass subsequently decreased but remained between approximately 0.47 and 0.55 g L^−1^ until the end of the experiment. In the presence of DCF, biomass production was lower. In glucose + DCF medium, biomass reached a maximum of approximately 0.23 g L^−1^ on day 2 and subsequently declined, reaching approximately 0.04 g L^−1^ on day 7. Addition of ammonium increased biomass production in the presence of DCF, reaching approximately 0.45 g L^−1^ on day 2 and maintaining higher biomass values than the glucose + DCF treatment throughout most of the incubation period.

DCF concentrations decreased progressively in both DCF-containing treatments. The initial DCF concentration decreased by approximately 67% by day 7 in the glucose + ammonium + DCF treatment. In the glucose + DCF treatment, initial DCF decreased by approximately 55% at the end of the experiment. Although some fluctuations occurred during the time course, particularly after day 3, the overall trend was a progressive reduction in DCF concentration. Notably, DCF continued to decrease after maximum biomass had been reached on day 2.

Such behavior is consistent with the marked strain dependence reported for bacterial DCF transformation. Some microorganisms can directly metabolize DCF; for example, Mohamed et al. [13] reported degradation efficiencies of 97.79, 88.72, 75.20 and 38.99% for *Achromobacter spanius* S11, *Achromobacter piechaudii* S18, *Pseudomonas aeruginosa* S1 and *Alcaligenes aquatilis* S2, respectively, when DCF was supplied at 40 mg L^−1^ as the sole carbon source. In contrast, other microorganisms show limited DCF transformation unless an additional growth substrate is supplied. For example, DCF removal by *Enterobacter hormaechei* D15 increased from approximately 52% in the absence of an external carbon source to 82% in the presence of glucose, while acetate supplementation substantially enhanced DCF transformation by *Brevibacterium* sp. D4 [11]. Similarly, *Labrys portucalensis* F11 completely degraded 6.8 mg L^−1^ DCF within six days under cometabolic conditions with acetate [21], while *Stenotrophomonas humi* strain DIC_5 was capable of degrading 75% of DCF at an initial concentration of 1.5 mg L^−1^ in the presence of glucose (3.0 g L^−1^) [22].

Nevertheless, DCF markedly affected bacterial growth: biomass production in the glucose + DCF condition remained substantially below that observed in the glucose control, indicating that the ability of Z2 to transform DCF does not imply the absence of a physiological effect of the pharmaceutical. Interestingly, ammonium supplementation partially restored biomass production in the presence of DCF, but this higher biomass did not translate into enhanced DCF removal. Ammonium may have favored biomass synthesis without stimulating DCF transformation, potentially due to changes in the carbon-to-nitrogen balance affecting the expression or activity of enzymes involved in this process.

This apparent uncoupling between growth and DCF removal is also evident from the temporal profiles. Maximum biomass was reached approximately on day 2, whereas DCF concentration continued to decrease during the subsequent period, when net biomass production had ceased or biomass was declining. Enzymatic activity maintained in non-growing cells, extracellular enzymatic activity, or metabolic processes expressed during late growth phases could potentially contribute to DCF transformation. However, elucidation of the enzymes involved would be required to discriminate among these possibilities.

Members of the genus *Streptomyces* possess extensive oxidative enzymatic capabilities. Of particular relevance, CYP105 family cytochromes from *Streptomyces* have been demonstrated to transform DCF: CYP105A1 from *Streptomyces griseolus* and CYP105D7 from *Streptomyces avermitilis* catalyze regioselective hydroxylation of DCF to 4′-hydroxydiclofenac [23,24]. Although the participation of CYP450 enzymes or other oxidative systems was not investigated in Z2 and therefore cannot be inferred from the present results, these reports provide a mechanistic basis for future investigation of DCF-transforming enzymes in this strain.

The removal capacity of Z2 is particularly noteworthy in the context of actinobacterial DCF biotransformation, for which comparatively few studies are available. Moreover, recent work with environmental bacteria belonging to *Rhodococcus* and *Kocuria* (both Actinomycetota) demonstrated DCF removal under glucose-supplemented conditions, although planktonic removal efficiencies remained below 35% of 10 mg L^−1^ DCF [25].

Further optimization of experimental conditions, including glucose and ammonium concentrations, inoculum size, pH, incubation time, and cell immobilization, could potentially enhance DCF removal beyond that achieved in this study.

Finally, the decrease in parent DCF concentration should be interpreted as bioremoval rather than complete biodegradation or mineralization, since TPs and mineralization were not assessed in the present study. This distinction is important because DCF can undergo multiple transformation pathways, including hydroxylation, oxidation, dechlorination, and cleavage reactions [13,26]. Thus, the present results demonstrate substantial removal of the parent compound by Z2, while further chemical characterization will be required to determine the fate of DCF and assess whether removal is accompanied by detoxification or complete biodegradation.

### 2.4. Biological Responses Depend on the Test Organism and Endpoint

#### 2.4.1. Plant-Based Ecotoxicological Assessment

Plant responses differed according to species, developmental endpoint, and composition of the exposure medium (Figure 4 and Figure S3). *Raphanus sativus* showed no consistent effects attributable to DCF on germination, radicle elongation, or hypocotyl elongation within the concentration range evaluated, whereas ammonium-containing media produced more evident changes in some endpoints. *Lycopersicon esculentum* showed greater differentiation among treatments, particularly for radicle elongation (Figure S3). The glucose + DCF medium reduced tomato radicle development relative to the corresponding control, whereas treatment with Z2 significantly increased radicle elongation. Notably, this condition corresponded to the highest DCF removal observed in the bioremoval assay.

The limited response of radish to DCF is consistent with the species- and endpoint-dependent sensitivity reported for pharmaceuticals and DCF. Previous studies have shown that germination may remain unaffected at concentrations that alter root development, while responses vary substantially among plant species [27,28,29].

The responses observed in ammonium-containing media indicate that the composition of the exposure matrix contributed to the phytotoxicological response. Excess NH_4_^+^ can affect plant development by altering pH and ion homeostasis, carbon and nitrogen metabolism, and root growth, with tomato being particularly sensitive to ammonium nutrition [30,31]. Therefore, changes observed in ammonium-containing treatments may reflect the effects of the nitrogen source rather than DCF alone. Other matrix components, including residual glucose and ammonium, salts, pH changes, and metabolites released during bacterial growth, may likewise influence seedling development. Consequently, matrix-matched controls are essential for distinguishing effects associated with residual DCF from those caused by the composition of the treated medium.

Particularly relevant was the improvement in tomato radicle elongation following treatment with Z2. Since root elongation can be a sensitive endpoint for DCF exposure [29], this response suggests that Z2 treatment may have generated conditions less inhibitory to early root development. This finding is especially relevant when considered together with the high DCF removal achieved by Z2, although the improved response cannot be attributed exclusively to DCF removal. Indeed, DCF transformation may generate products with biological activities different from those of the parent compound [32]. Therefore, the favorable radicle response provides complementary biological evidence suggesting an improvement in the quality of the treated medium and highlights the value of combining chemical removal measurements with ecotoxicological endpoints when assessing bioremediation effectiveness.

#### 2.4.2. Earthworm Responses

Exposure to the different DCF-containing media significantly affected both body-mass loss and coelomocyte abundance in *E. fetida* (Figure 5 and Figure S4). Earthworms exposed to untreated glucose + DCF and glucose + ammonium + DCF media showed body-mass losses of approximately 0.043 and 0.045 g, respectively. Following Z2 treatment, body-mass loss decreased to approximately 0.010 g in the glucose + DCF + Z2 condition, whereas it remained similar to the value observed in the corresponding control in the glucose + ammonium + DCF + Z2 condition.

Coelomocyte abundance was markedly higher in the ammonium-containing media. Earthworms exposed to glucose + DCF showed approximately 6.3 × 10^6^ cells mL^−1^, whereas those exposed to glucose + ammonium + DCF showed approximately 9.8 × 10^6^ cells mL^−1^. Following Z2 treatment, coelomocyte abundance remained similar, reaching approximately 6.2 × 10^6^ cells mL^−1^ in glucose + DCF + Z2 and 9.3 × 10^6^ cells mL^−1^ in glucose + ammonium + DCF + Z2. No significant differences were observed relative to the corresponding controls.

The response of *E. fetida* is environmentally relevant because DCF is bioavailable to earthworms. Carter et al. [33] demonstrated DCF uptake by *E. fetida* and *Lumbricus terrestris* following exposure to contaminated soil. More recently, DCF exposure in *E. fetida* was associated with effects on growth and reproduction and with metabolic and transcriptional alterations related to energy metabolism and physiological homeostasis [34]. These findings support the use of sublethal endpoints such as body-mass variation to evaluate the biological effects of DCF-containing media.

The marked reduction in body-mass loss following Z2 treatment in the glucose + NH_4_^+^ + DCF condition is consistent with a lower physiological disturbance in earthworms. However, this effect cannot be attributed exclusively to DCF removal because the organisms were exposed to the complete culture media, which also contained residual nutrients, microbial metabolites and other matrix components. Interestingly, this reduction was not observed in the ammonium-containing medium, where body-mass loss remained similar after Z2 treatment. This difference indicates that the biological response was influenced by the composition of the exposure medium rather than by DCF removal alone.

Coelomocytes are immunocompetent cells involved in the cellular defense of earthworms and are responsive to environmental contaminants. Irizar et al. [35] showed that major coelomocyte populations, including amoebocytes and eleocytes, can differ in their sensitivity to toxicants. The higher abundance observed in the ammonium-containing media may therefore reflect an effect of the exposure matrix on the coelomic cellular response. However, total coelomocyte abundance alone does not allow the underlying mechanism to be established, since changes in cell number may result from alterations in viability, mobilization or the relative abundance of different cell types.

The earthworm results complement the plant bioassays. In tomato, the glucose + DCF + Z2 treatment produced the most favorable radicle response. In contrast, the ammonium-containing Z2 treatment did not reduce earthworm body-mass loss relative to its control, although ammonium was associated with higher coelomocyte abundance.

This divergence may reflect differences in the composition of the treated media, including residual DCF, nutrients, bacterial metabolites, and potentially DCF TPs. Therefore, the DCF removal does not necessarily imply a proportional reduction in toxicity. Further characterization of TPs would help clarify their contribution to the observed biological effects.

Differences in experimental matrices, DCF concentrations, and analytical approaches should also be considered when comparing these findings with previous studies (Table 1). Lara-Moreno et al. [32] evaluated activated sludge isolates and consortia, whereas Aguilar-Romero et al. [36] investigated complex biopurification systems, where sorption and contaminant interactions may influence DCF dissipation. Furthermore, differences in ecotoxicological endpoints highlight the importance of evaluating biological responses alongside chemical removal. In this context, the present study provides complementary evidence from plant and earthworm assays, emphasizing the relevance of both DCF removal and residual medium composition in determining biological responses.

## 3. Materials and Methods

### 3.1. Microorganisms and Chemicals

Actinobacterial strains were obtained from the culture collection of the Actinobacteria Biotechnology Laboratory at PROIMI-CONICET (Tucumán, Argentina). The panel comprised *Amycolatopsis tucumanensis* AB0 and *Streptomyces* spp. A5, ER1, MC1, M7, SG1, SG21, Z2, and Z38. These microorganisms were selected because previous studies demonstrated their ability to tolerate, remove, or detoxify environmental contaminants [37,38]. *Streptomyces* spp. E5N50 and E7N12 were obtained from the culture collection of the Institute for Integrative Biology of the Cell (Gif-sur-Yvette, France).

Pharmaceutical-grade diclofenac sodium (Saporiti AR. Buenos Aires, Argentina) was used in the tolerance and removal assays. A 10 g L^−1^ stock solution was prepared in waterand stored at 4 °C.

### 3.2. Diclofenac Tolerance Screening

Tolerance was evaluated on starch-casein agar (SCA) plates containing 10.0 g starch, 1.0 g casein, 0.5 g K_2_HPO_4_, and 12.0 g agar per liter (pH 7.0) (Sigma-Aldrich, Darmstadt, Germany). Rectangular troughs were cut in the center of each plate and filled with DCF solutions of 1, 5, or 10 g L^−1^. Control plates contained no DCF and were otherwise treated identically [39].

Each strain was inoculated onto the agar surface by streaking a spore suspension perpendicular to the trough. Plates were incubated at 30 °C for 7 d. Growth, sporulation, and spore pigmentation were visually evaluated and assigned individual scores ranging from 0 to 3. For growth, a score of 0 indicated no visible growth, 1 indicated growth restricted to the first third of the streak, closest to the inoculation edge, 2 indicated growth extending over approximately two-thirds of the streak, and 3 indicated growth throughout the entire streak, comparable to the DCF-free control. For sporulation and spore pigmentation, a score of 0 indicated their absence, whereas scores of 1 and 2 indicated partial responses restricted to specific colony regions (e.g., edges or center), and 3 indicated sporulation or pigmentation throughout the entire growth area. The three scores were summed to obtain a composite tolerance score ranging from 0 to 9, which was used to visualize strain- and concentration-dependent response patterns in a heatmap. Scores were assigned based on direct visual examination of the cultures, as sporulation and spore pigmentation were not always clearly distinguishable in photographs.

### 3.3. Diclofenac Removal Assays

#### 3.3.1. Inoculum Preparation

The selected strains were pre-cultured in 100-mL Erlenmeyer flasks containing 30 mL Tryptic Soy Broth medium (TSB) (Laboratorios Britania S.A., Buenos Aires, Argentina) and incubated at 30 °C and 180 rpm for 72 h. Biomass was recovered by centrifugation (7500 rpm, 20 min, 4 °C), washed three times with sterile distilled water, and resuspended in sterile distilled water. To reduce the influence of intracellular reserves and standardize the physiological state, washed biomass was incubated in sterile distilled water at 30 °C and 180 rpm for 12 h. The biomass was recovered again and resuspended to 100 g wet weight L^−1^ [39].

#### 3.3.2. Experimental Design

Removal assays were conducted in 125-mL flasks containing 30 mL minimal medium composed of 0.5 g L^−1^ K_2_HPO_4_, 0.2 g L^−1^ MgSO_4_·7H_2_O, and 0.01 g L^−1^ FeSO_4_·7H_2_O. DCF (nominally 50 mg L^−1^), glucose (1.0 g L^−1^), and ammonium sulfate (4 g L^−1^) (Sigma-Aldrich, Darmstadt, Germany) were supplied in combinations designed to test DCF in the absence or presence of conventional carbon and nitrogen sources. Corresponding DCF-free controls and abiotic controls without biomass were processed under identical conditions. Flasks were inoculated to a final concentration of 2 g wet biomass L^−1^ and incubated at 30 °C and 180 rpm for 120 h.

Six independent biological replicates were prepared for each experimental condition. Three replicates were used for dry biomass determination and analysis of the corresponding culture supernatants (glucose and ammonium), while the remaining three were used for DCF quantification. For DCF analysis, the entire culture (supernatant and bacterial biomass) was subjected to extraction to account for both dissolved and biomass-associated DCF. Uninoculated controls were included for each experimental condition to assess potential abiotic DCF removal.

#### 3.3.3. Growth and Removal Kinetics

The time-course assay was performed using the strain and culture conditions selected based on the results of the endpoint assay. Cultures were incubated in 125-mL flasks containing 30 mL of minimal medium at 30 °C and 180 rpm for 7 d. Samples were analyzed at 24-h intervals. Three biological replicates were used for dry-biomass determination and glucose and ammonium quantification in the corresponding culture supernatants, while the remaining three replicates were processed as whole cultures (biomass and supernatant) for DCF extraction and quantification. A separate set of flasks was harvested at each sampling time; therefore, cultures were not repeatedly sampled, and no withdrawn volume was replaced. Inoculum concentration, medium composition, and the remaining incubation conditions were identical to those used in the endpoint assay.

### 3.4. Sample Processing and Analytical Determinations

Upon completion of the incubation period, the entire culture volume (30 mL) from each replicate flask designated for chemical analysis was processed as a single sample for DCF extraction. Whole-culture extraction, including both the aqueous phase and biomass, was performed to quantify the total recoverable residual DCF and to minimize the possibility that adsorption to biomass, association with extracellular polymeric substances, or cellular uptake would be misinterpreted as DCF transformation. After extraction, residual DCF was quantified by HPLC-UV using a Shimadzu NEXERA XR UHPLC system equipped with a PDA detector (Shimad-zu Corporation, Kyoto, Japan), following the previously published analytical method described by Velazquez Yañez et al. [25], which reports the chromatographic conditions and method validation parameters are reported in detail.

Three additional biological replicates were used to determine biomass production and residual glucose and ammonium concentrations. Cultures were centrifuged at 7500 rpm for 20 min at 4 °C to separate the biomass from the supernatant. Biomass pellets were transferred to pre-weighed aluminum containers and dried at 105 °C to constant weight. Biomass production was expressed as grams of dry cell weight per liter of culture medium.

Glucose concentration in the recovered supernatants was determined using a commercial Glucose (HK) Assay Kit (Wiener lab^®^, Buenos Aires, Argentina), whereas ammonium concentration was determined using the Uremia Assay Kit (Wiener lab^®^, Buenos Aires, Argentina). Glucose and ammonium consumption were calculated relative to their corresponding initial concentrations.

To ensure comparability among treatments, inoculated biotic assays and uninoculated abiotic controls were subjected to the same incubation, extraction, and analytical procedures. DCF removal was calculated using the residual concentration measured in the corresponding abiotic control as the reference.

### 3.5. Ecotoxicological Assessment

#### 3.5.1. Plant Bioassays

Tomato (*Lycopersicon esculentum*) and radish (*Raphanus sativus*) were used as bioindicators of DCF toxicity. Germination, radicle elongation, and hypocotyl elongation were assessed.

Petri dishes were lined with filter paper moistened with 3 mL of test solution. A second filter paper was placed over the first, and seeds were distributed uniformly: 20 tomato seeds or 10 radish seeds per dish. Dishes were incubated in darkness at 22 ± 2 °C for 168 h. Germination was expressed as:

Germination (%) = (number of germinated seeds/total number of seeds) × 100.

Radicle and hypocotyl lengths were measured with ImageJ 1.53k (National Institutes of Health, Bethesda, MD, USA). The radicle was measured from its point of emergence to the root apex, and the hypocotyl from the seedling collar to the cotyledon insertion point [40]. The assay was performed using three independent biological replicates per condition. For each replicate, three plates were prepared from the same culture, resulting in three technical pseudoreplicates per replicate. Measurements from the three plates were averaged to obtain a single value for each biological replicate (*n* = 3 per condition).

#### 3.5.2. Earthworm Bioassays

The ecotoxicological effects of the contaminants on *Eisenia fetida* were assessed using weight loss and coelomocyte concentration as biomarkers.

Healthy, adult, clitellated *Eisenia fetida* (180–350 mg) were obtained from Compostuc (Tucumán, Argentina) and maintained at 22 ± 2 °C and approximately 60% relative humidity, with weekly feeding of compost and plant-derived material. Prior to exposure, earthworms were placed on moist paper in darkness for 24 h to allow gut clearance. They were then rinsed with sterile distilled water, gently blotted on absorbent paper, and individually weighed in Petri dishes to determine their initial body mass. After 3 days of exposure, surviving earthworms were processed in the same manner and weighed again to determine body-mass loss.

The assays were performed following the methodology described by Aparicio et al. [40], using 5 earthworms per replicate and three independent replicates per experimental condition (15 earthworms per treatment). After 3 days of exposure at 22 ± 2 °C, mortality was assessed by gentle mechanical stimulation, and surviving earthworms were individually weighed to determine body-mass loss.

Coelomocytes were collected by electrical stimulation at 9 V in 1 mL of phosphate-buffered saline (PBS) containing 0.02% EDTA. Cell suspensions were centrifuged at 1500 rpm for 10 min at 4 °C, washed, resuspended in PBS (pH 7.0 ± 0.2), and counted using a Neubauer chamber. Cell concentrations were expressed as cells mL^−1^.

### 3.6. Statistical Analysis

All experiments were performed using three independent biological replicates per treatment (n = 3). When technical replicates were included, their measurements were averaged to obtain a single value for each biological replicate before statistical analysis. Results are expressed as mean ± standard deviation (SD). Statistical analyses were performed using Minitab^®^ Statistical Software, version 17.2.1 (Minitab Inc., State College, PA, USA). Normality was assessed using the Shapiro–Wilk test, and homogeneity of variances was evaluated using Bartlett’s test. When these assumptions were met, differences among treatments for each quantitative response variable were evaluated using one-way analysis of variance (ANOVA), followed by Tukey’s honestly significant difference (HSD) test for multiple comparisons. Differences were considered statistically significant at *p* < 0.05.

## 4. Conclusions

This study demonstrates the potential of actinobacteria, particularly *Streptomyces* sp. Z2, for DCF bioremoval under glucose-supplemented conditions. *Streptomyces* sp. Z2 combined high phenotypic tolerance with a strong capacity to remove DCF, reaching 90.8% removal in glucose-supplemented medium, while nutrient availability modulated the balance between biomass production and contaminant removal. The kinetic profiles suggest a possible contribution of non-growth-associated DCF removal mechanisms, including cometabolism, which requires further confirmation.

The ecotoxicological assays added an important functional dimension to the chemical results. *Streptomyces* sp. Z2 treatment was associated with improved responses in some biological endpoints, although the condition associated with the highest DCF removal was not uniformly the most favorable across test organisms. Tomato radicle elongation improved under glucose + DCF + Z2, whereas glucose + ammonium + DCF + Z2 produced the lowest earthworm body-mass loss. These complementary responses show that removal efficiency and biological effects are related but distinct dimensions of remediation performance.

Overall, the integrated approach used here supports *Streptomyces* sp. Z2 as a promising candidate for the development of DCF bioremediation strategies and highlights the value of combining contaminant quantification with ecotoxicological endpoints.

Future studies should identify DCF TPs by LC-MS/MS, assess their persistence and ecotoxicity, and investigate the relative contributions of biosorption and enzymatic degradation, including potential oxidative and hydrolytic pathways. Further work could optimize nutrient conditions and evaluate *Streptomyces* sp. Z2 in soil and wastewater systems, including repeated-batch and immobilized-cell approaches.

## Figures and Tables

**Figure 1:**
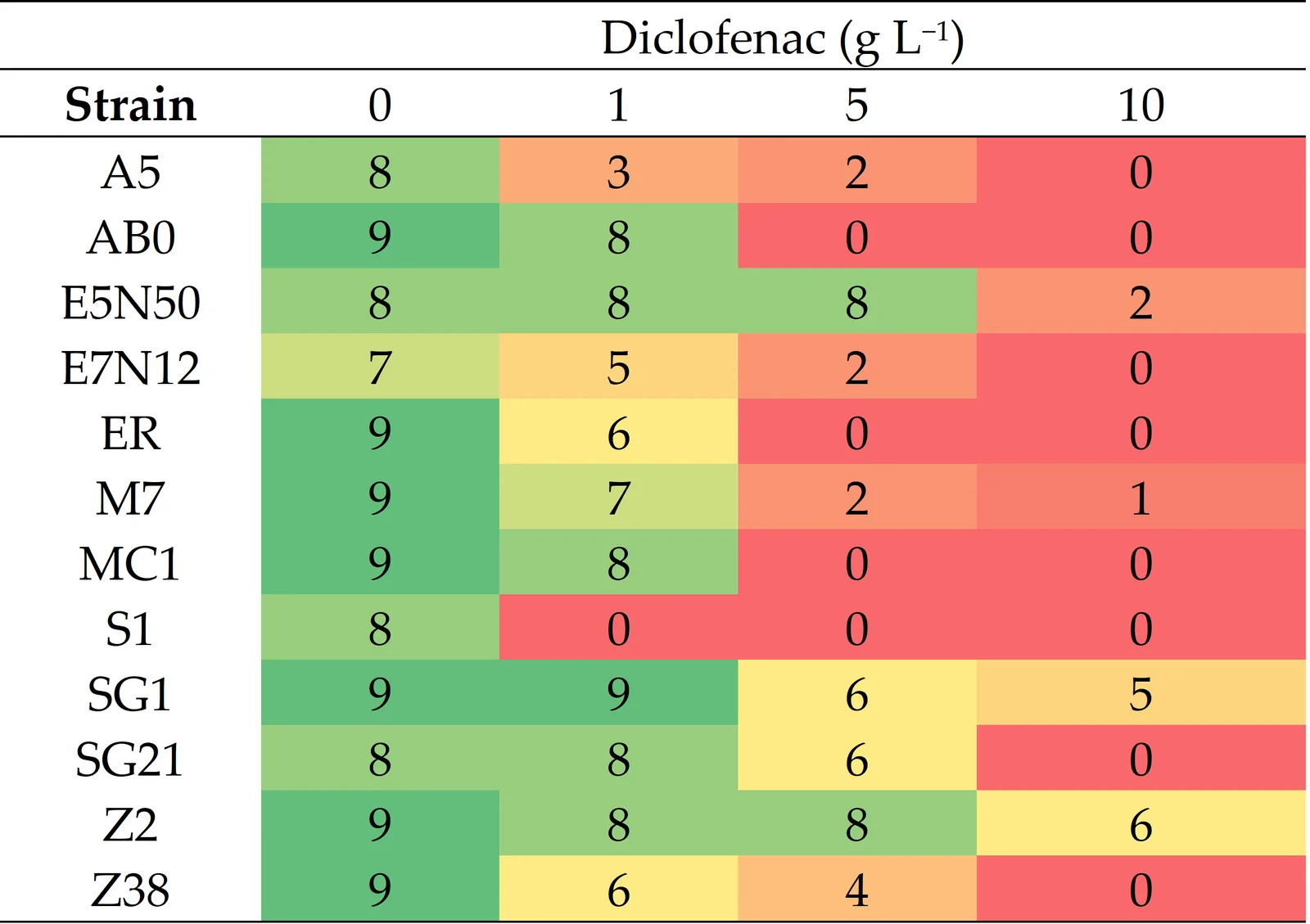
Heatmap of composite phenotypic tolerance scores of actinobacterial strains exposed to different DCF concentrations. Scores range from 0 to 9 and represent the sum of growth, sporulation, and spore pigmentation scores (0 to 3 for each parameter).

**Figure 2:**
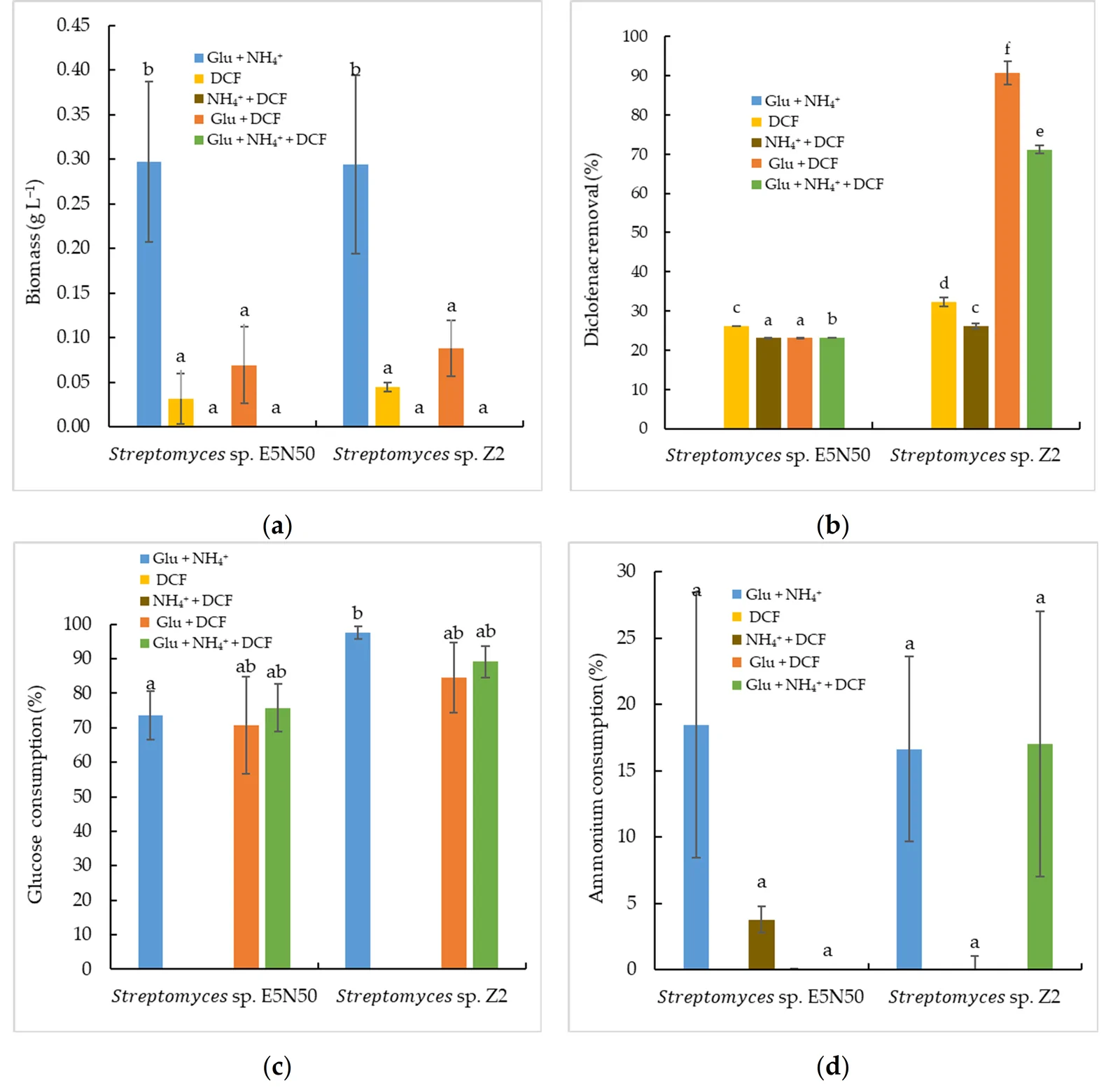
Biomass production (**a**), DCF removal (**b**), glucose consumption (**c**), ammonium consumption (**d**) by *Streptomyces* spp. E5N50 and Z2 after 120 h, under different carbon and nitrogen conditions. Within each graph, different letters indicate significant differences between means (*p* < 0.05).

**Figure 3:**
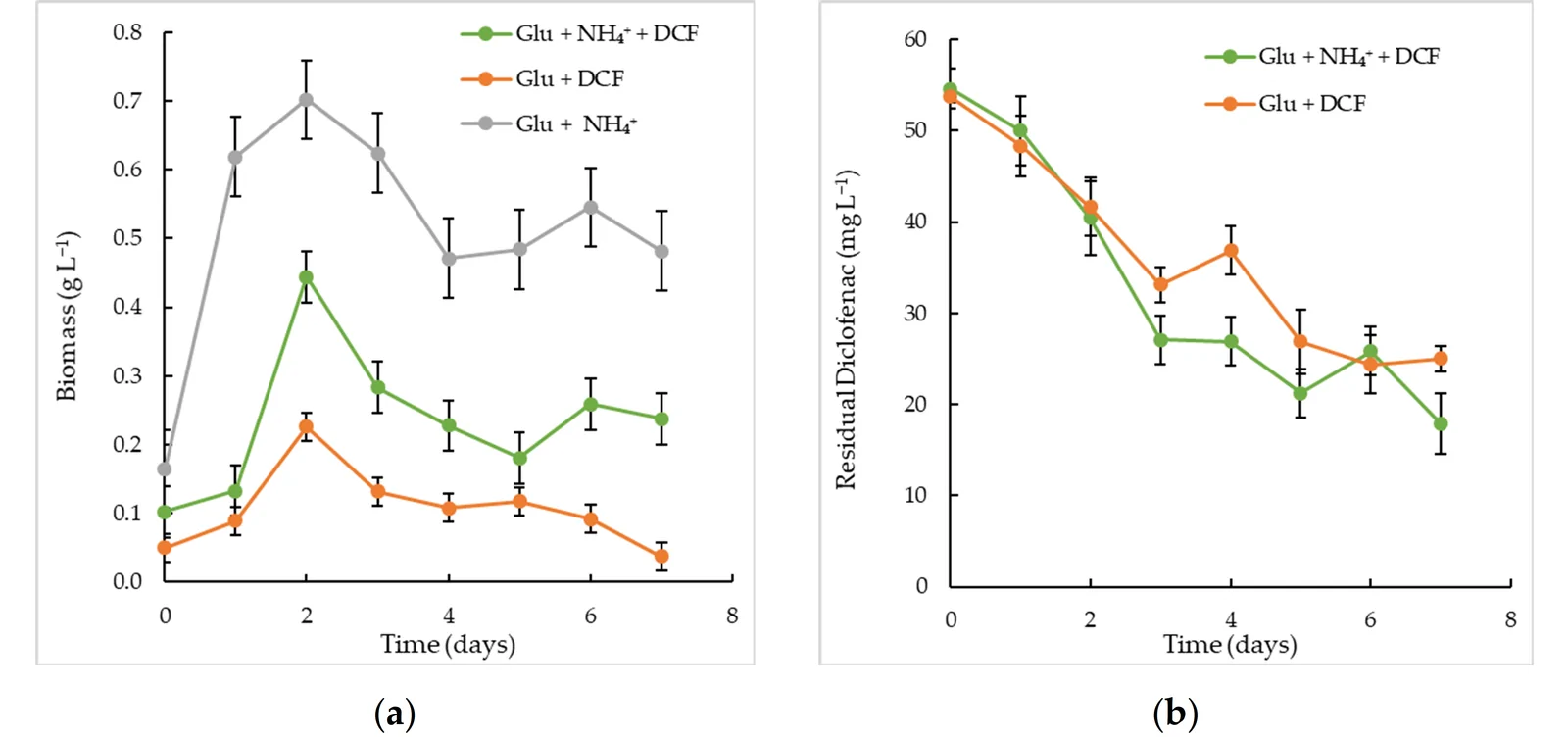

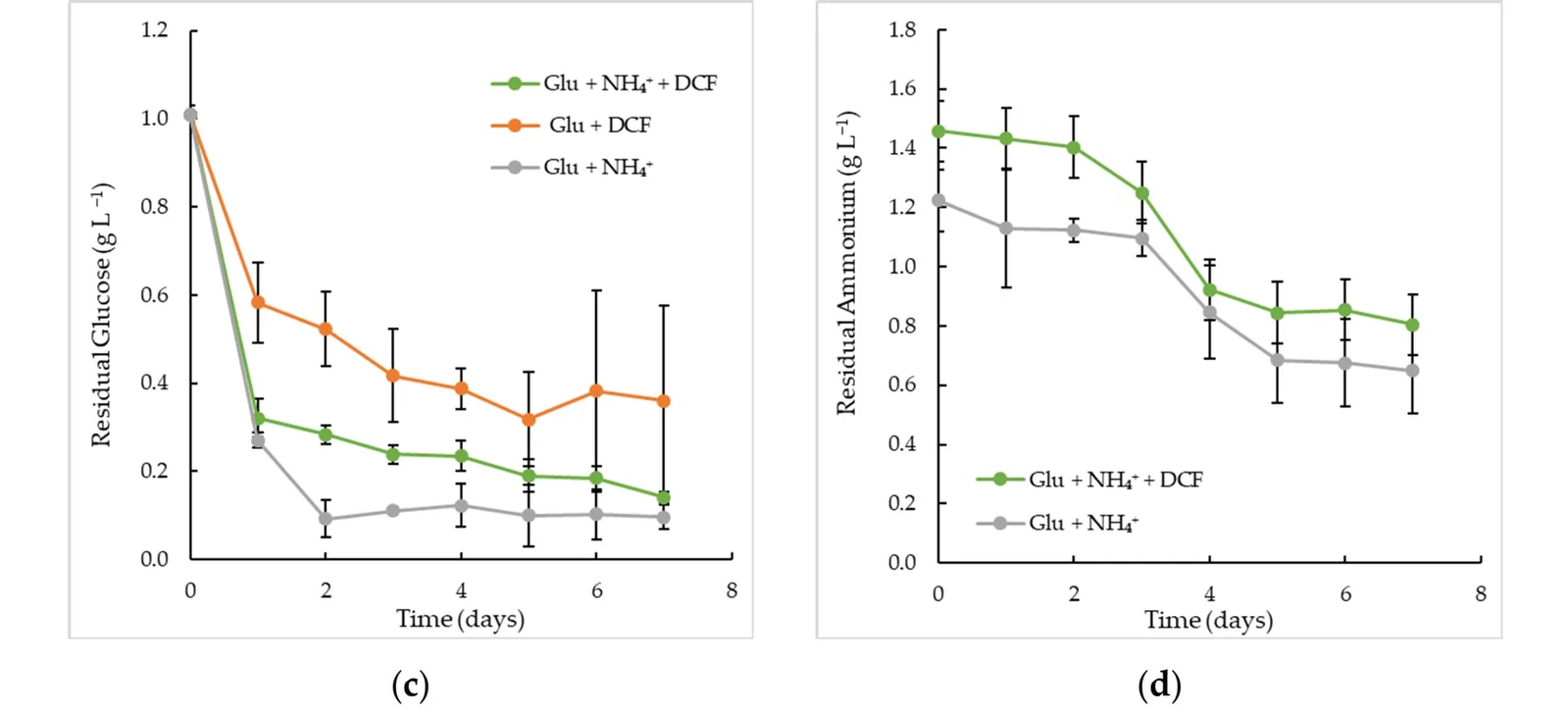
Time courses of biomass, glucose, ammonium, and DCF in *Streptomyces* sp. Z2 cultures: (**a**) biomass production; (**b**) residual diclofenac; (**c**) residual glucose; (**d**) residual ammonium. Data are presented as mean ± SD.

**Figure 4:**
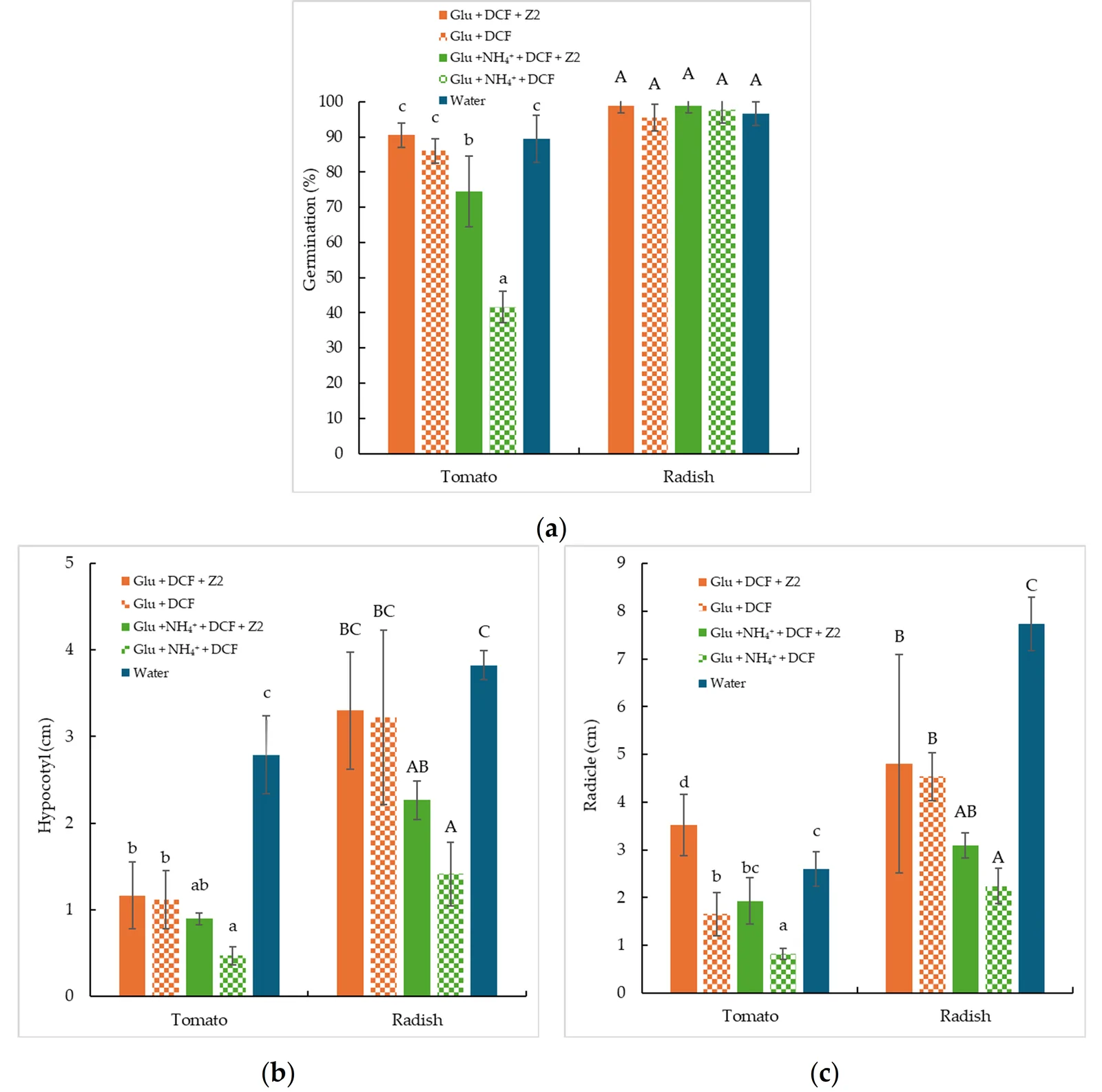
Seedling-growth responses of tomato and radish exposed to DCF and *Streptomyces* sp. Z2-treated media. (**a**) Germination. (**b**) Hypocotyl length. (**c**) Radicle length. Within each graph, different letters indicate significant differences between means (*p* < 0.05). Uppercase and lowercase letters are compared separately, with comparisons made only among letters of the same case.

**Figure 5:**
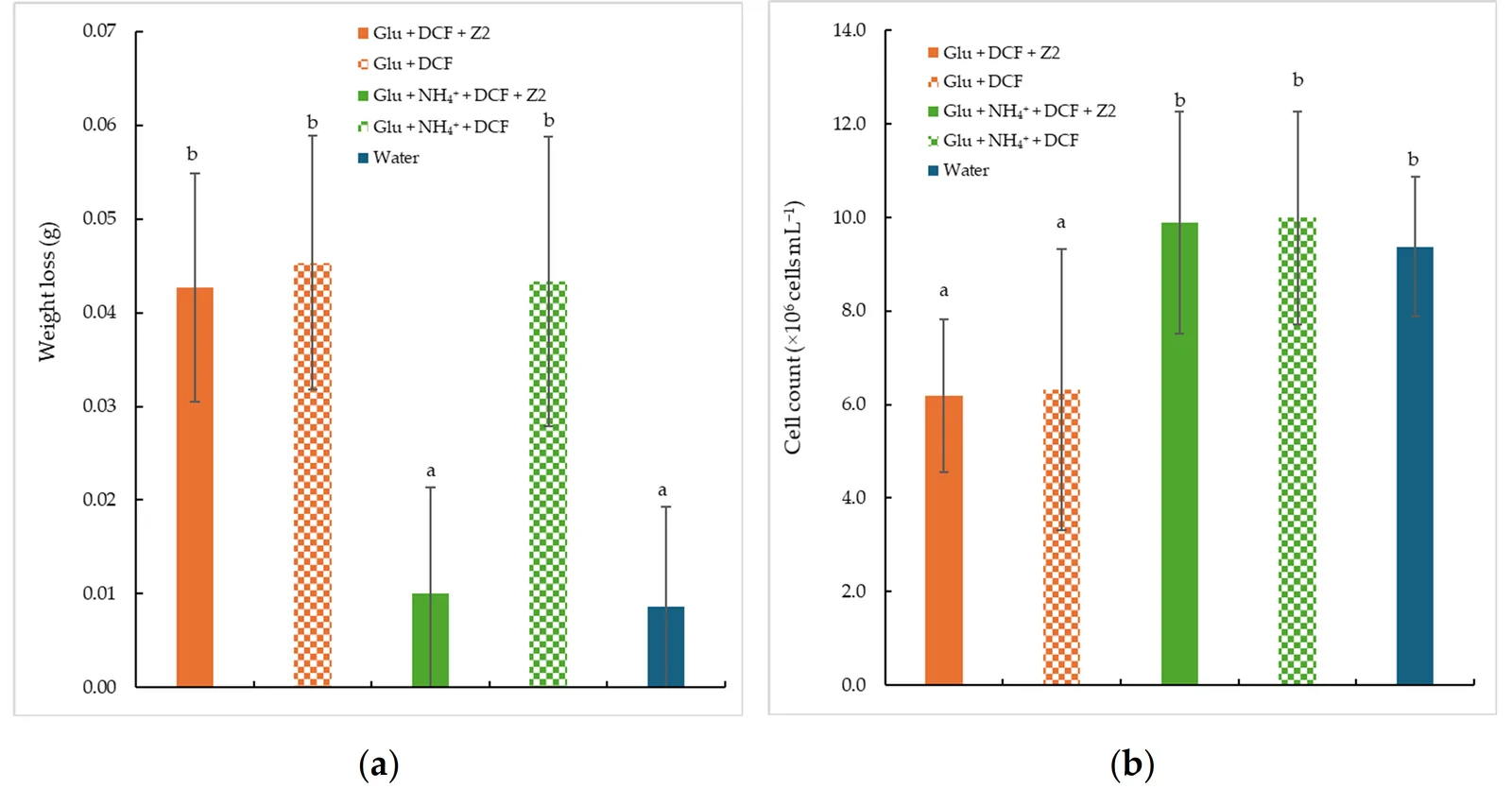
Body-mass change (**a**) and coelomocyte concentration (**b**) in *Eisenia fetida* exposed to DCF and *Streptomyces* sp. Z2-treated media. Within each graph, different letters indicate significant differences between means (*p* < 0.05).

**Table 1:** Comparison of experimental approaches, diclofenac removal assessments, and ecotoxicological endpoints in the present study and previous investigations.

Feature/Criterion	Present Study	Lara-Moreno et al. [32]	Aguilar-Romero et al. [36]
**Contaminant(s) Investigated**	DCF alone (50 mg L^−1^ in liquid assays; phenotypic screening up to 10 g L^−1^).	DCF alone (10 mg L^−1^ in biodegradation assays; 24 and 200 mg L^−1^ in enrichments).	Multi-compound mixture of emerging contaminants: Ibuprofen (IBP, 100 µg g^−1^), DCF, 20 µg g^−1^), and Triclosan (TCS, 20 µg g^−1^), tested individually and in mixtures.
**Biological Agent(s) Used**	Defined actinobacterial strains (*Streptomyces* spp. E5N50 and Z2), highlighting *Streptomyces* sp. Z2 as a defined biocatalyst under cometabolic conditions.	Isolated bacterial strains (*Pseudomonas aeruginosa* CSWD.1, *Pseudomonas* sp. CSWD.2) and a mixed microbial consortium (MC) isolated from activated sludge.	Indigenous microbial communities residing in biopurification system (BPS/biobed) microcosms composed of soil, vermicompost, and olive tree prunings.
**Analytical/Chemical Endpoints**	Biomass production, glucose and ammonium consumption, and parent DCF bioremoval via whole-culture extraction followed by HPLC-UV.	DCF concentration by HPLC-ODS, LC-MS/MS metabolite profiling (4′-OH-DCF, 5-OH-DCF, NO_2_-DCF/TP339), kinetic modeling (SFO/HS), IC_50_, and 16S/ITS metabarcoding.	QuEChERS extraction followed by HPLC dissipation kinetics, 16S rRNA gene amplicon sequencing, qPCR quantification of key taxa, and LEfSe biomarker discovery.
**Ecotoxicological Assessment**	Terrestrial bioassays evaluating plant seedling growth (*Lycopersicon esculentum* and *Raphanus sativus*: germination, radicle, hypocotyl) and earthworm responses (*Eisenia fetida*: body-mass loss, coelomocyte abundance).	Aquatic bioluminescence assay using *Aliivibrio fischeri*/Microtox® Test System (Microbics Corporation, Carlsbad, CA, USA) to evaluate pre- and post-biodegradation samples.	Soil bacterial viability via LIVE/DEAD^®^ BacLight™ fluorescence microscopy and Neubauer cell counts in biomixture samples.
**Main Novelty & Contribution**	Demonstrates high DCF bioremoval (up to 90.8%) by defined *Streptomyces* strains, integrated with terrestrial bioassays to assess changes in the biological effects of treated media on plants and earthworms.	Establishes a metabolite-focused pathway linking persistent TPs (4′-OH-DCF, NO_2_-DCF) to increased post-treatment aquatic ecotoxicity, supported by metabarcoding of *Burkholderia* and *Talaromyces* dynamics.	Evaluates soil-based biopurification systems for multi-compound PPCP dissipation, identifying high community resilience and specific bacterial biomarkers in agricultural waste biomixtures.

## Data Availability

The original contributions presented in this study are included in the article/Supplementary Materials. Further inquiries can be directed to the corresponding author.

## Supplementary Materials

The following supporting information can be downloaded at https://www.mdpi.com/article/10.3390/ijms27219952/s1.

## Abbreviations

The following abbreviations are used in this manuscript:

DCF	Diclofenac
Glu	Glucose
NH_4_^+^	Ammonium
TPs	Transformation products
HPLC-UV	High-Performance Liquid Chromatography with Ultraviolet Detection
PBS	Phosphate-buffered saline
EDTA	Ethylenediaminetetraacetic acid
SD	Standard deviation
