# Supplementary material for: Diclofenac Removal by Actinobacterial Strains and Ecotoxicological Responses
Source: Int J Mol Sci. 2026 Nov 6;27(21):9952. doi: 10.3390/ijms27219952 (PMC13764526; doi:10.3390/ijms27219952)
Supplement: Supplementary file 1 [file ijms-27-09952-s001.zip › Figure S1.pdf]

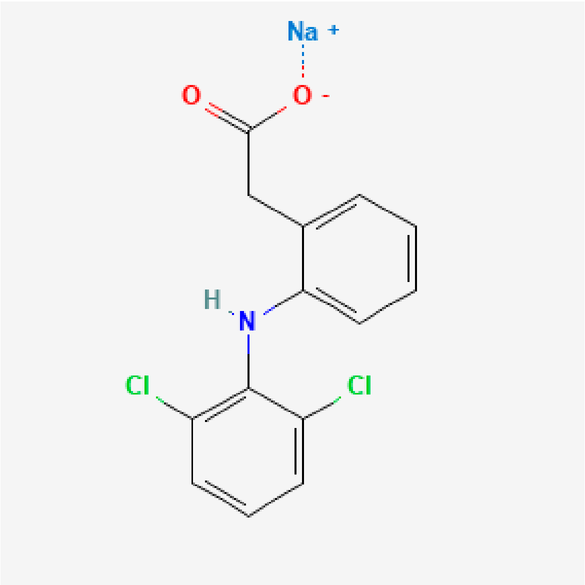

Figure S1: Chemical structure of diclofenac sodium. National Center for Biotechnology Information (2026). PubChem Compound Summary for CID 5018304, Diclofenac sodium. Retrieved September 30, 2026 from https://pubchem.ncbi.nlm.nih.gov/compound/Diclofenac-sodium.
