# Supplementary material for: Diclofenac Removal by Actinobacterial Strains and Ecotoxicological Responses
Source: Int J Mol Sci. 2026 Nov 6;27(21):9952. doi: 10.3390/ijms27219952 (PMC13764526; doi:10.3390/ijms27219952)
Supplement: Supplementary file 1 [file ijms-27-09952-s001.zip › Figure S2.pdf]

DCF 0 g L^{-1}	10 g L^{-1}	5 g L^{-1}	1 g L^{-1}

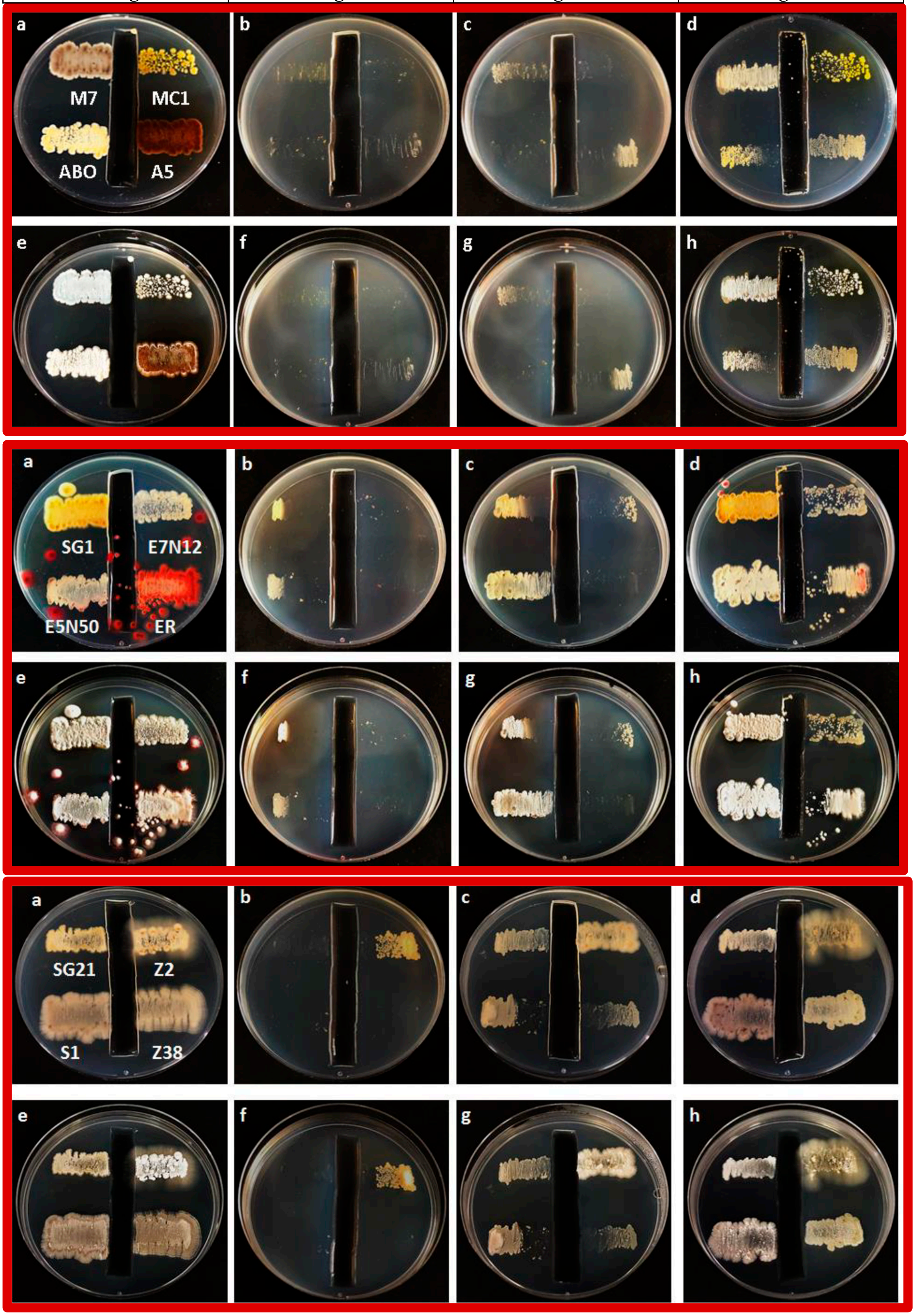

Figure S2: Diclofenac Tolerance Screening on SCA, using DCF 0, 1, 5 and 10 g L^{-1}. (a-d) Petri dish Front; (e-f) Petri dish Back.
