# Supplementary material for: Diclofenac Removal by Actinobacterial Strains and Ecotoxicological Responses
Source: Int J Mol Sci. 2026 Nov 6;27(21):9952. doi: 10.3390/ijms27219952 (PMC13764526; doi:10.3390/ijms27219952)
Supplement: Supplementary file 1 [file ijms-27-09952-s001.zip › Figure S3.pdf]

(A)

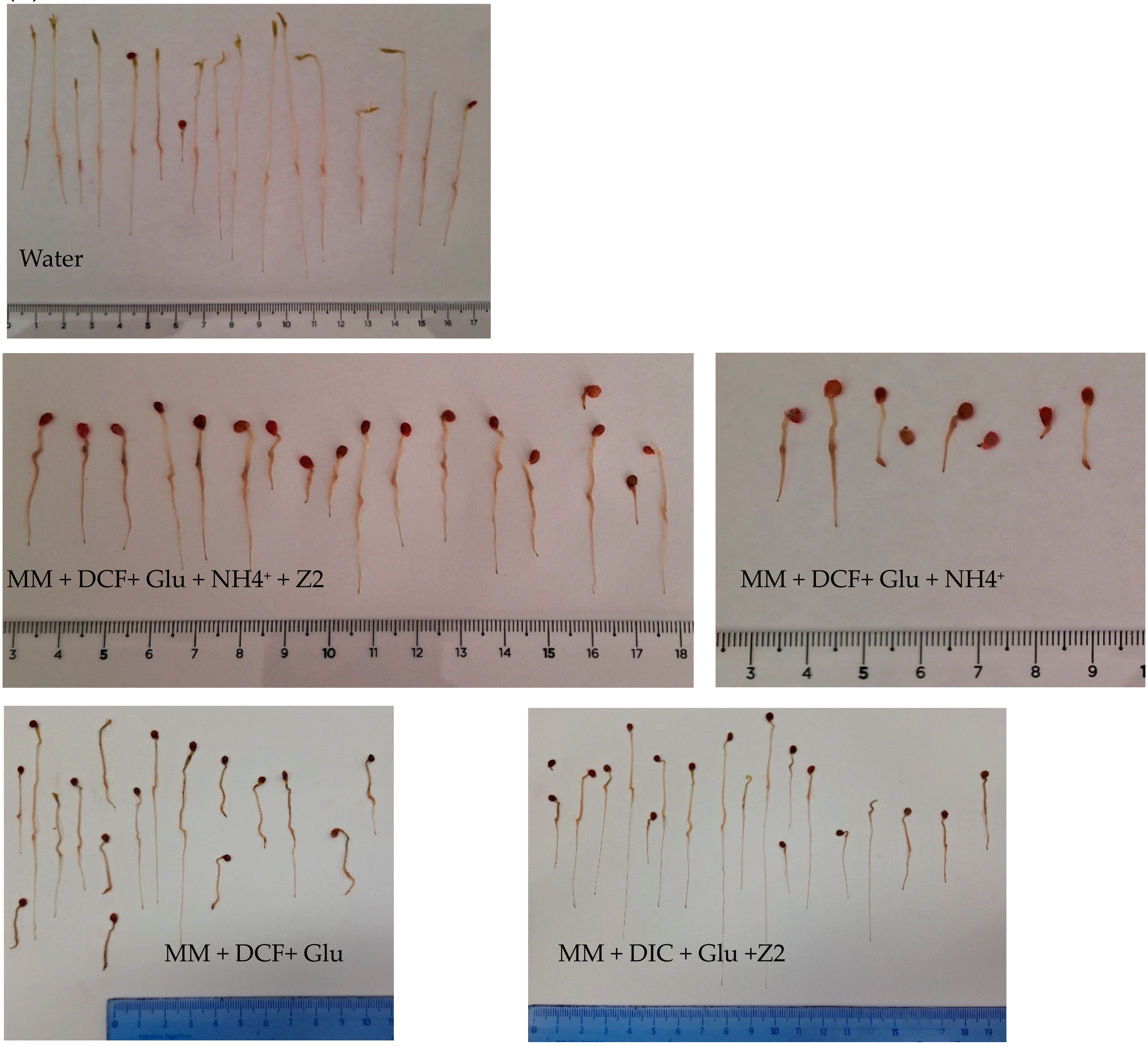

(B)

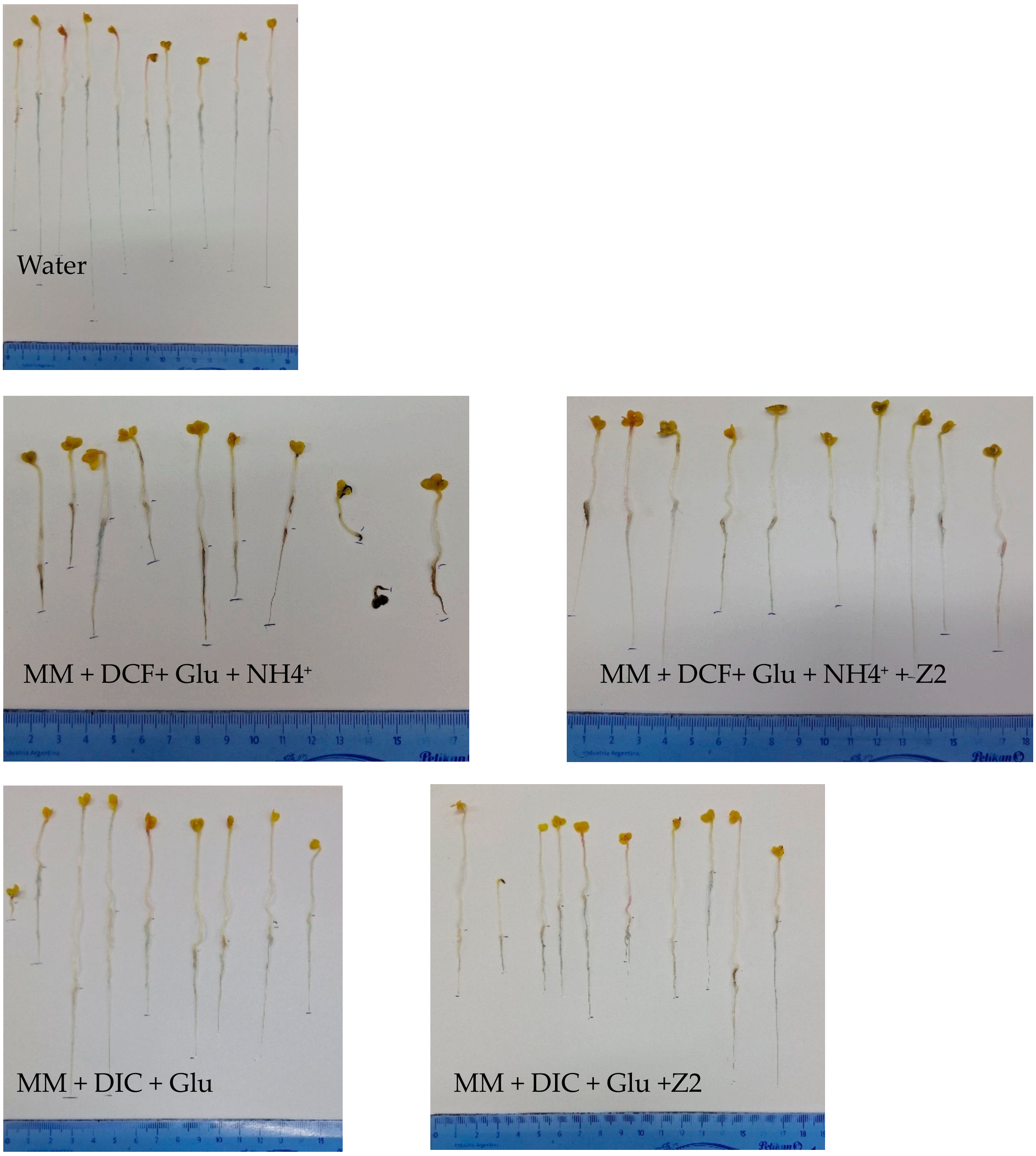

Figure S3. Representative photographs of (A) tomato (*Solanum lycopersicum*) and (B) radish (*Raphanus sativus*) seedlings exposed to the different experimental treatments. Images illustrate seedling development and the appearance of radicles and hypocotyls under the evaluated conditions. Treatment identities and scale bars are indicated in each panel.
