# Supplementary material for: Diclofenac Removal by Actinobacterial Strains and Ecotoxicological Responses
Source: Int J Mol Sci. 2026 Nov 6;27(21):9952. doi: 10.3390/ijms27219952 (PMC13764526; doi:10.3390/ijms27219952)
Supplement: Supplementary file 1 [file ijms-27-09952-s001.zip › Figure S4.pdf]

Water

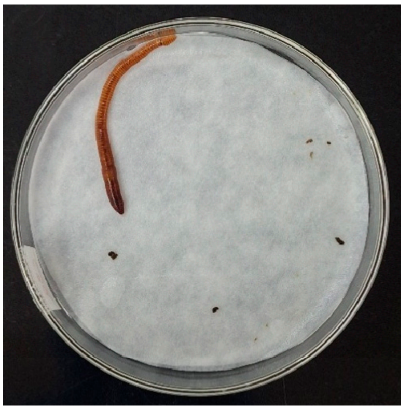

MM + DCF+ Glu + $NH4^{+}$

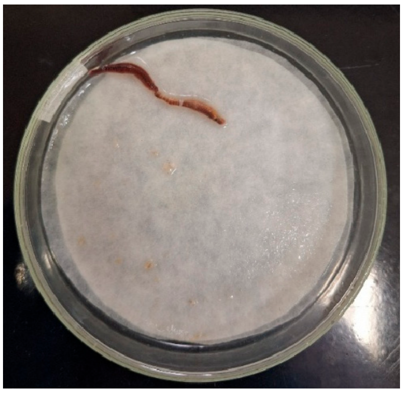

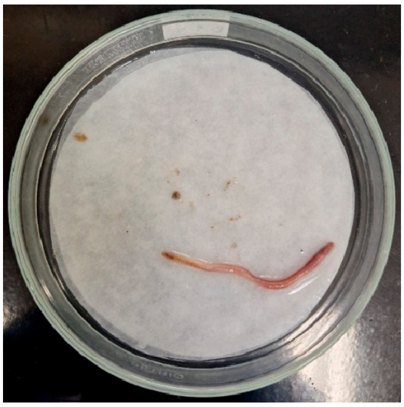

MM + DCF+ Glu + Z2

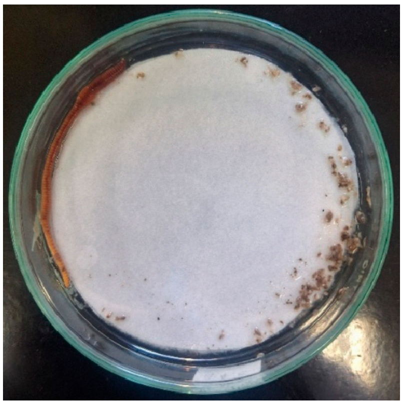

Figure S4. Representative photographs of *Eisenia fetida* specimens after 3 days of exposure to the experimental treatments. Images show earthworms exposed to the water control and three selected treatments in which visible morphological alterations were observed. No relevant macroscopic changes were observed in the remaining treatments; therefore, photographs of those conditions are not included. Treatment identities are indicated in each panel.
